# Adaptation of Enterovirus 71 to Adult Interferon Deficient Mice

**DOI:** 10.1371/journal.pone.0059501

**Published:** 2013-03-19

**Authors:** Elizabeth A. Caine, Charalambos D. Partidos, Joseph D. Santangelo, Jorge E. Osorio

**Affiliations:** 1 Department of Pathobiological Sciences, School of Veterinary Medicine, University of Wisconsin-Madison, Madison, Wisconsin, United States of America; 2 Inviragen Inc., Madison, Wisconsin, United States of America; 3 Inviragen Inc., Singapore, Singapore; University of Texas Medical Branch, United States of America

## Abstract

Non-polio enteroviruses, including enterovirus 71 (EV71), have caused severe and fatal cases of hand, foot and mouth disease (HFMD) in the Asia-Pacific region. The development of a vaccine or antiviral against these pathogens has been hampered by the lack of a reliable small animal model. In this study, a mouse adapted EV71 strain was produced by conducting serial passages through A129 (α/β interferon (IFN) receptor deficient) and AG129 (α/β, γ IFN receptor deficient) mice. A B2 sub genotype of EV71 was inoculated intraperitoneally (i.p.) into neonatal AG129 mice and brain-harvested virus was subsequently passaged through 12 and 15 day-old A129 mice. When tested in 10 week-old AG129 mice, this adapted strain produced 100% lethality with clinical signs including limb paralysis, eye irritation, loss of balance, and death. This virus caused only 17% mortality in same age A129 mice, confirming that in the absence of a functional IFN response, adult AG129 mice are susceptible to infection by adapted EV71 isolates. Subsequent studies in adult AG129 and young A129 mice with the adapted EV71 virus examined the efficacy of an inactivated EV71 candidate vaccine and determined the role of humoral immunity in protection. Passive transfer of rabbit immune sera raised against the EV71 vaccine provided protection in a dose dependent manner in 15 day-old A129 mice. Intramuscular injections (i.m.) in five week-old AG129 mice with the alum adjuvanted vaccine also provided protection against the mouse adapted homologous strain. No clinical signs of disease or mortality were observed in vaccinated animals, which received a prime-and-boost, whereas 71% of control animals were euthanized after exhibiting systemic clinical signs (P<0.05). The development of this animal model will facilitate studies on EV71 pathogenesis, antiviral testing, the evaluation of immunogenicity and efficacy of vaccine candidates, and has the potential to establish correlates of protection studies.

## Introduction

Hand, foot and mouth disease (HFMD) is an emerging human-viral disease causing significant public health concerns across the Asia-Pacific region. The disease affects mostly children and is characterized by ulcers and vesicles on the hands, feet and oral cavity [1], [2]. In some instances, neurological manifestations are observed including aseptic meningitis, brainstem encephalitis, pulmonary edema, and polio-like paralysis [1], [2], [3]. In 2010, a HFMD outbreak in China caused at least 1.7 million cases and 905 deaths [4]. In 2011, severe outbreaks were reported in many Asian countries including Japan (346,000 cases), and Vietnam (110,000 cases and 160 deaths) [5]. It has also been reported in Singapore, Europe, Australia, Middle East and the United States [2]. Seroprevalence and seroincidence has been documented in high disease burden countries to better understand EV71 pathogenesis [6], [7]. Epidemiological studies in the beginning of 2012 have shown the number of HFMD cases rising in China and Singapore, remaining steady in Japan and Vietnam, and causing a recent outbreak in Cambodia [5], [8].

HFMD is caused by viruses which belong to the Enterovirus genus in the *Picornaviridae* family. These viruses are non-enveloped, positive sense RNA viruses that include polio, coxsackie, echo, and other enteroviruses [2]. While many different enteroviruses are associated with HFMD, EV71 has been one of the main causative agents over the past decade. EV71 has also been isolated from the most severe cases of HFMD and is associated with neurological disease and death [9], [10]. Unfortunately, there is no vaccine or an antiviral currently available for EV71 and the development has been hindered due to the lack of a reliable small animal model.

Animal models provide a means to study viral pathogenesis and host immune responses to infection [11], [12], [13], [14], [15]. In addition, they can allow for the testing of vaccine and antiviral candidates and in establishing immune correlates of protection. While adult mice (>6 weeks-old) are resistant to EV71 infection [2], recent studies have shown the susceptibility of neonatal outbred and interferon (IFN) deficient mice to EV71 for up to two weeks post birth [13], [14], [15], [16]. Consequently, the immaturity of their immune system and short window for which they are susceptible to disease limits their use for vaccine efficacy studies.

AG129 (α/β and γ IFN receptor deficient) mice can sustain infection of many viruses including dengue, Sindbis virus, and rhesus rotavirus [14]. INFs are essential for the initial nonspecific host defenses against many viruses and promote the onset of various immune responses [17]. The lack of functional IFNs in AG129 mice, support the systemic spread and persistence of viruses that don’t normally use mice as a natural host and helps establish infection. Despite IFN deficiency, AG129 mice are known to produce effective humoral and cellular immune responses [11]. An AG129 mouse model has been used to demonstrate the safety and efficacy of dengue vaccine candidates, showing the usefulness of this model in testing potential vaccines for EV71 [12].

Here we report the successful generation of a mouse adapted EV71 strain by conducting serial passages through A129 (α/β IFN receptor deficient) and AG129 mice. This adapted strain produced systemic clinical signs of polio-like disease in three month-old AG129 mice that included limb paralysis, hunched back, eye irritation, loss of balance, and death. Our results also highlighted the role of IFN-γ in controlling EV71 infection since adult A129 mice that lack IFN α/β receptors were resistant to infection. Furthermore, passive immunization with rabbit EV71 immune sera and active immunization with an alum adjuvanted inactivated EV71 candidate vaccine provided full protection against EV71 disease using this model. This vaccine has currently completed phase I clinical trials.

## Materials and Methods

### 2.1 Viruses and Cell Culture

An EV71 B2 isolate, MS/7423/87 (Genbank: U22522.1), was obtained from Inviragen, Inc. (Singapore, Singapore). This virus was selected to prepare an EV71 candidate vaccine on the basis of amino acid sequence similarity to highly immunogenic strains as well as high yield in Vero cell culture [unpublished data]. Virus was grown at a multiplicity of infection (MOI) of 0.001 in Vero cells (ATCC CCL-81) in Dulbecco’s modified minimal essential medium (DMEM) containing 2% FBS and penicillin-streptomycin. Once the cells displayed 80% cytopathic effect (CPE), two freeze thaw cycles were completed, followed by centrifugation at 2000×g for 15 minutes at 4°C to remove cellular debris. Viral supernatants were harvested and stored in aliquots at −80°C. The 50% tissue culture infective dose (TCID_50_) was determined in Vero cells using the Reed and Muench formula [18].

### 2.2 Ethics Statement

Treatment of animals was done in accordance with the University of Wisconsin-Madison Standard Operating Procedures, which follows closely with the regulations outlined in the USDA Animal Welfare Act and the Guide for the Care and Use of Laboratory Animals. All animal experiments were approved by the University of Wisconsin Institutional Animal Care and Use Committee (IACUC) (Protocol #: V01458).

### 2.3 Mouse Adaptation

One day-old AG129 mice (B & K Universal Ltd., UK) (n = 4) received intraperitoneal (i.p.) injections of 10^6^ TCID_50_/ml (in 50 µl volume) of the parental (non-adapted) B2 sub-genotype of EV71. Mice showing clinical signs of EV71 infection (neurological signs, weight loss) were euthanized. Brains were aseptically removed and homogenized as 10% suspensions (w/v) in phosphate buffered saline (PBS) that contained 0.1% bovine serum albumin (BSA). Tissue suspensions were centrifuged at 2,300×g for 20 minutes at 4°C. Supernatants were separated into aliquots and stored at −80°C and designated as passage one (P1). This P1 EV71 virus was injected i.p. (100 µl) into groups of 12 day-old A129 mice (n = 6). Viral stocks were prepared using the same method as described above and designated as passage two (P2). A third and final passage (P3) was conducted by i.p. injections (100 µl) of P2 virus into 15 day-old A129 mice (n = 7). Equivalent passages were also completed with control mice (n = 3) of the same strain and age using a 10% brain homogenate from uninfected AG129 mice. To prepare viral stocks of the mouse adapted EV71 strain, P3 virus was propagated once through Vero cells and stored in aliquots at −80°C. This is referred to as the mouse adapted EV71 strain.

### 2.4 EV71 Detection by RT-PCR

Total RNA was extracted from each viral passage, including the final propagation of the mouse adapted strain, using QIamp viral RNA minikit (Qiagen, Valencia, CA), as per the manufacturer’s instructions. RNA samples were tested for the presence of EV71 by RT-PCR using a One-Step RT-PCR Kit (Qiagen, Valencia, CA). Primers (sequences available upon request) were used to amplify a 2.7 kb region including the 5′ untranslated region (UTR) and capsid proteins (VP1–VP4). Positive (EV71 parental virus) and negative (uninfected mouse brain, distilled water) controls were also included. PCR products were visualized by agarose gel electrophoresis.

### 2.5 Mouse Studies

The mouse adapted strain was tested in a comparative study between A129 and AG129 mice. Groups of 10 week-old AG129 and A129 mice (n = 5–6) were given i.p. injections of 1.3×10^5^ TCID_50_/ml (in 400 µl volume) of the parental or mouse adapted EV71 strains. Control mice were injected by the same route with PBS. Mice were monitored for three weeks for clinical signs of disease, weight loss, and mortality.

A passive transfer study was performed using four groups (n = 4) of 15 day-old A129 mice. Groups of mice were injected i.p. with 200 µl of either neat, 10-, or 100-fold dilution of rabbit immune sera raised against the EV71 inactivated vaccine with a titer of 1∶524,288. A control group received PBS (same volume and route). After 24 hours all mice were challenged i.p. with 1.1×10^4^ TCID_50_ units/ml (in 200 µl volume) of the mouse adapted EV71 strain.

An active immunization study was performed using the EV71 inactivated candidate vaccine. Two groups of five week-old AG129 mice (n = 7) were injected intramuscularly (i.m.) with a preparation containing 3 µg of inactivated EV71 virus and 10 mg/ml of alum (in 100 µl volume). Previous studies in mice have shown that this amount of the inactivated virus was highly immunogenic in mice [unpublished data].The alum adjuvanted vaccine preparations were mixed in PBS and rocked at 4°C for 2 hours before vaccination. A negative control group (n = 7) received PBS injections (same volume and route). On day 21 post prime, one group of EV71 vaccinated mice received an identical booster injection while the second group of immunized mice and negative controls received PBS (same volume and route). Serum neutralizing antibody responses against EV71 were measured in individual blood samples collected on days 21 and 33 post prime. On day 35 post prime, all groups of mice were challenged i.p. with 1.3×10^5^ TCID_50_/ml of the mouse adapted strain. Animals were monitored twice a day for three weeks for clinical signs of disease, weight loss, and mortality. Serum samples were collected on days 3 and 7 post challenge and tested for viremia by RT-PCR. Individual serum samples were also collected on day 28 post challenge to determine neutralizing antibody titers to EV71.

### 2.6 Histopathology

Immediately after death or euthanasia of mice, tissue samples of brain, heart, lung, kidney, spleen and intestine were collected from vaccinated, positive control, and negative control animals and fixed in 10% neutral buffered formalin. Skulls were decalcified in a 10% EDTA solution. Tissues and heads were paraffin embedded, sectioned and stained with hematoxylin and eosin (H&E).

### 2.7 Determination of Viremia Levels in Infected Mice by Real Time RT-PCR

Viremia levels were tested in serum samples collected on days 3 and 7 post challenge during the active immunization study. A SYBR Green real-time RT-PCR assay was performed to estimate virus concentration (TCID_50_/ml). Viral RNA was isolated from serum using a QiaAmp Viral RNA kit (Qiagen, Valencia, CA). Total RNA was extracted from 60 µl of sample and eluted in a final volume of 60 µl of elution buffer. Primers (sequences available upon request) were designed targeting a 100 bp region in the VP1 gene. A QuantiTect SYBR Green RT-PCR kit (Qiagen, Valencia, CA) was used for the assay on a Bio-Rad iCylcer IQ5 thermal cycler (Bio-Rad, Hercules, CA). A standard curve was used to quantify the viral nucleic acid in each serum sample. The standard curve was generated from serially diluted samples of mouse adapted EV71 virus stock. A curve correlation coefficient of 0.995 and a PCR efficiency between 90–100% was used to validate the assay.

### 2.8 Neutralization Assays

Serum samples collected for neutralization assays were heat treated at 56°C for 30 minutes immediately before use to inactivate complement and other adventitious agents. The heat inactivated serum samples were tested for neutralizing antibodies against the EV71 parental B2 sub-genotype strain using a TCID_50_ neutralization assay in Vero cells. Each serum sample was tested in duplicate. Neutralization titer was defined as the dilution of serum required to neutralize ≥50% of the virus infected wells. Titer of less than ten was considered negative and given an inverse titer of one for calculation purposes. Data was expressed as geometric mean titers (GMT) which was calculated for each group of animals.

### 2.9 Statistical Analysis

Data from animal studies were compiled in Microsoft Excel and analyzed using Excel or Prism 5 (GraphPad, Inc.). Experiments including weight loss, viremia, and log-transformed reciprocal neutralizing antibody titers were analyzed using a Student’s *t* test [19]. A Fisher’s Exact Test was used for survival data. A two tailed P≤0.05 was considered significant.

## Results

### 3.1 Adaptation of EV71 Virus in Adult AG129 Mice

**Figure 1:**
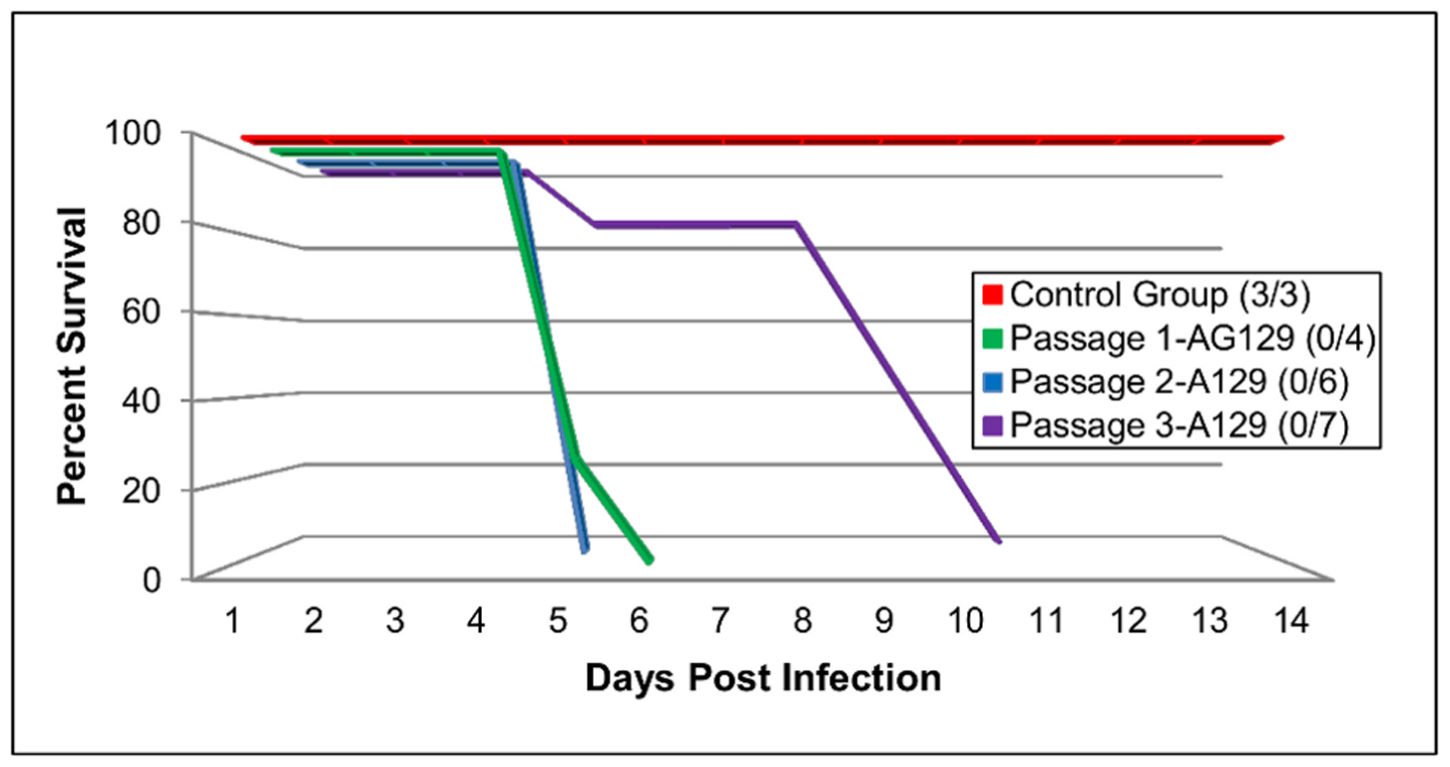
Survival of mice during serial passage of a B2 sub-genotype of EV71. A group of AG129 neonates received i.p. injections of 5×10^5^ TCID_50_/ml of the parental strain of EV71. This was designated as passage 1 (P1). Passage 2 (P2) was a group of 12 day-old A129 mice which received i.p. injections of 100µl of P1. Passage 3 (P3) was a group of 15 day-old A129 mice which received i.p. injections of 100 ul of P2. Each one of the serial passages has a group of the same strain of mice used as control and received PBS via the same route.

**Figure 2:**
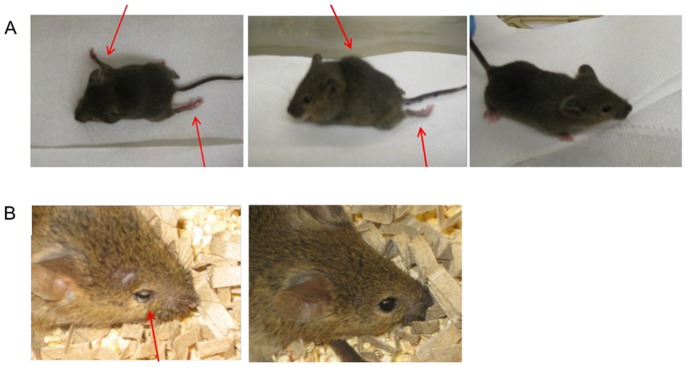
Clinical signs in A129 and AG129 mice following challenge with mouse adapted strain of EV71. A: 15 day-old A129 mice displaying hind/fore leg paralysis, hunched posture after i.p. injections with 100 µl of a brain homogenate prepared from 12 day-old A129 mice during adaptation process of EV71. The far right is a mouse from the control group which received a non infected mouse brain homogentate (same route and volume). **B:** 10 week-old AG129 mouse displaying eye irritation after i.p. injections with 1.3×10^5^ TCID_50_/ml of the mouse adapted strain of EV71. Right picture is a control mouse which received PBS (same route and volume).

Blind serial passages of EV71 MS/7423/87 resulted in the generation of an adapted virus that was lethal in adult AG129 mice. Initial inoculation of the EV71 parental strain by the i.p route with 50 µl of 5×10^5^ TCID_50_/ml in 1 day-old AG129 mice resulted in systemic disease and all inoculated animals (n = 4) were euthanized by day five post infection (Fig. 1). Inoculation of the P1 EV71 virus into 12 day-old A129 mice (n = 6) also resulted in 100% lethality by day three post-infection (p.i.) (Fig. 1). Interestingly, longer time was required to develop clinical signs after injection of the P2 virus into 15 day-old A129 mice (n = 7). It took nine days p.i. before all animals shown clinical signs and had to be euthanized. A trial inoculation of the P3 EV71 virus in 10 week-old AG129 mice (n = 4) resulted in clinical disease with three mice being euthanized between days 10–12 p.i., and one mouse showing no signs of disease (data not shown). Clinical signs observed in mice inoculated with passaged EV71 viruses included limb paralysis, hunched back, eye irritation, loss of balance, loss of movement control, and death (Fig. 2). Virus specific primers detected EV71 in the brain homogenates after each passage of the virus (data not shown).

### 3.2 Infection Studies in Adult A129 and AG129 Mice

**Figure 3:**
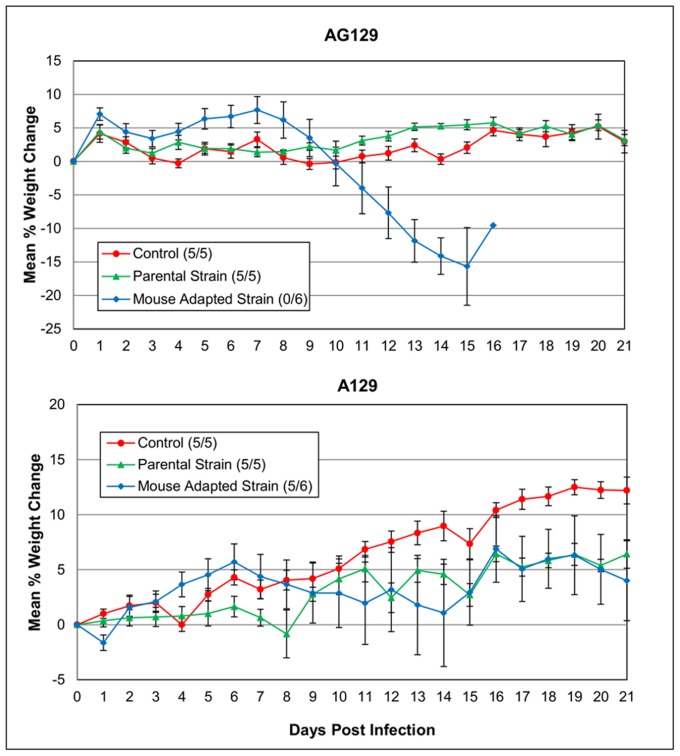
Mean percent weight change of interferon deficient AG129 and A129 mice used in comparison study. Groups of A129 and AG129 mice received i.p. injections of 1.3×10^5^ TCID_50_/ml of either the mouse adapted strain or parental strain of a B2 sub-genotype of EV71. Control mice received PBS (same route and volume). Weights and clinical signs of disease were taken daily for each mouse.

**Figure 4:**
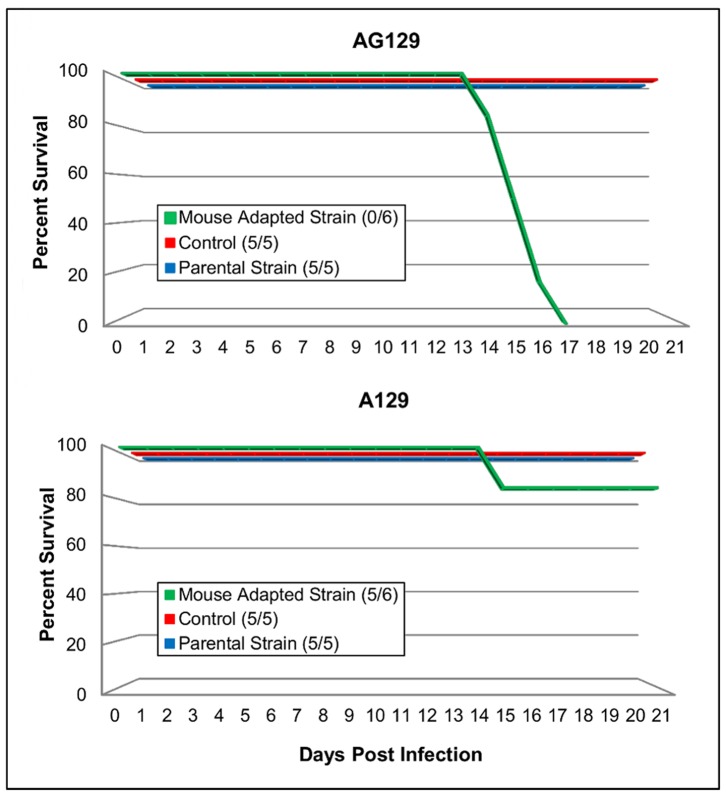
Percent survival between interferon deficient AG129 and A129 mice during comparison study. Groups of A129 and AG129 mice were given i.p. injections of 1.3×10^5^ TCID_50_/ml of either the mouse adapted strain or parental strain of a B2 sub-genotype of EV71. Control mice received PBS (same route and volume). After the onset of clinical signs of disease, mice were euthanized.

To determine the role of IFN in protection against EV71 viruses, the parental and mouse adapted EV71 viral strains were inoculated i.p. with 1.3×10^5^ TCID_50_/ml into groups of adult 10 week-old A129 and AG129 mice. The mouse adapted EV71 strain resulted in substantial clinical signs of disease in AG129 mice including limb paralysis, eye irritation, loss of balance and control of movements. Significant weight loss was also observed and the infection resulted in 100% mortality by day 17 p.i. (Fig. 3). In contrast, only one out of six A129 mice displayed clinical signs and died 14 day p.i. (same route and dose) suggesting that IFN-γ is sufficient in controlling virus replication (Fig. 4). This was statistically significant when compared to the same age AG129 mice (P<0.05). Statistically significant differences in weight loss were also detected on days 12–16 post challenge between AG129 mice receiving the mouse adapted strain and those inoculated with the parental virus and PBS (control) (P≤0.05). Inoculation of the EV71 parental strain (same route and dose) into both adult A129 and AG129 mice did not result in any clinical signs of infection indicating the importance of virus adaptation for increased susceptibility of infection. In addition, all animals either maintained or gained weight over the period of the study (Fig. 3).

### 3.3 Vaccine Protection Studies

**Figure 5:**
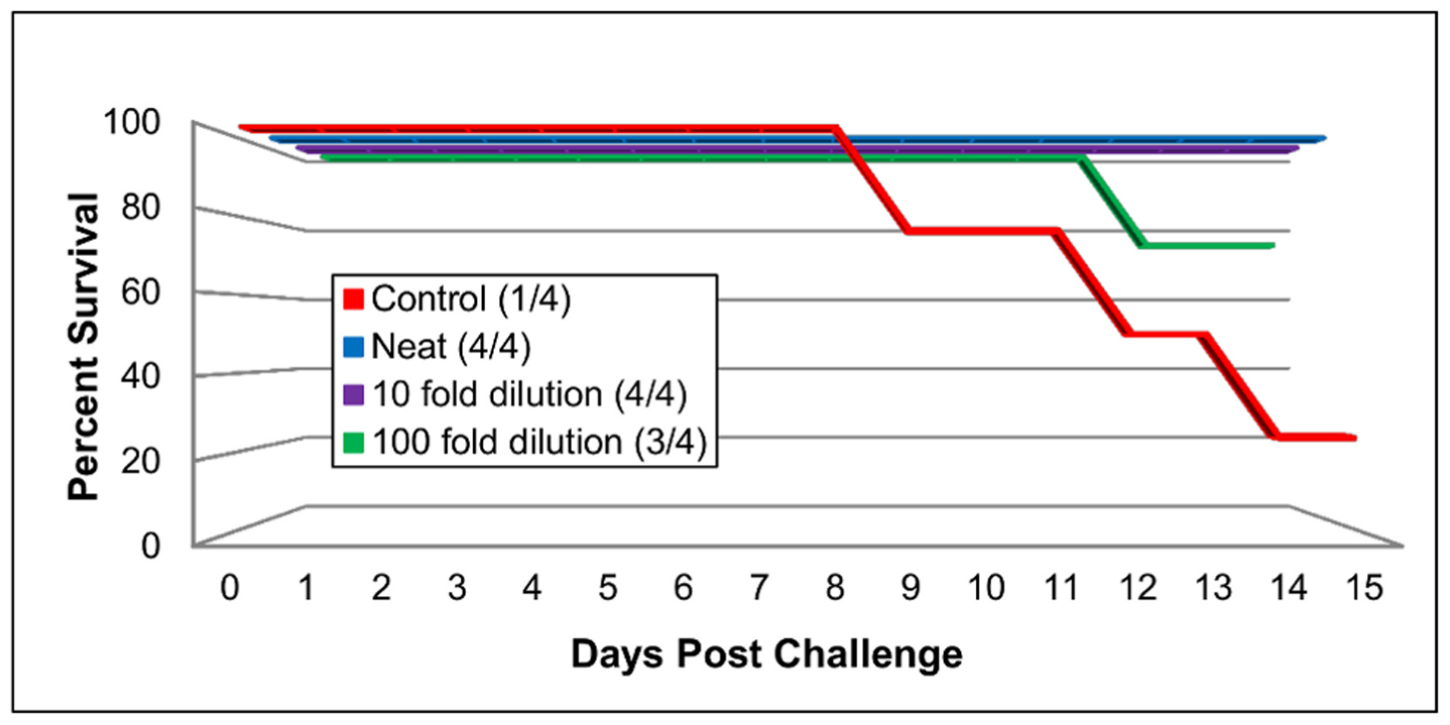
Percent survival of 15 day-old A129 mice in passive transfer study using EV71 neutralizing serum. 15 day-old IFN deficient A129 mice received i.p. injections of 200 ul of differing concentration of EV71 neutralizing rabbit serum. The 50% neutralizing titer of the serum was 1∶524288 (19 log_2_). Control mice received PBS (same route and volume). After 24 hours all mice were challenged i.p. with 1.1×10^4^ TCID50 units/ml of mouse adapted EV71. The mice were monitored daily for clinical signs of disease.

Efficacy studies were conducted in A129 and AG129 mice with the mouse adapted EV71 virus to determine the protective role of antibodies and vaccines against disease. Since young A129 mice are susceptible to infection a passive transfer study was conducted in groups of 15 day-old A129 mice (n = 4) using various dilutions of rabbit immune sera to the EV71 inactivated vaccine. After being challenged i.p. with the mouse adapted strain (24 h post passive immunization), all mice that received neat and 10-fold diluted sera showed no clinical signs of disease and survived (Fig. 5). Three out of four mice that received 100-fold dilution sera also survived the challenge. In contrast, in the control group three out of four mice showed clinical signs and were euthanized.

**Figure 6:**
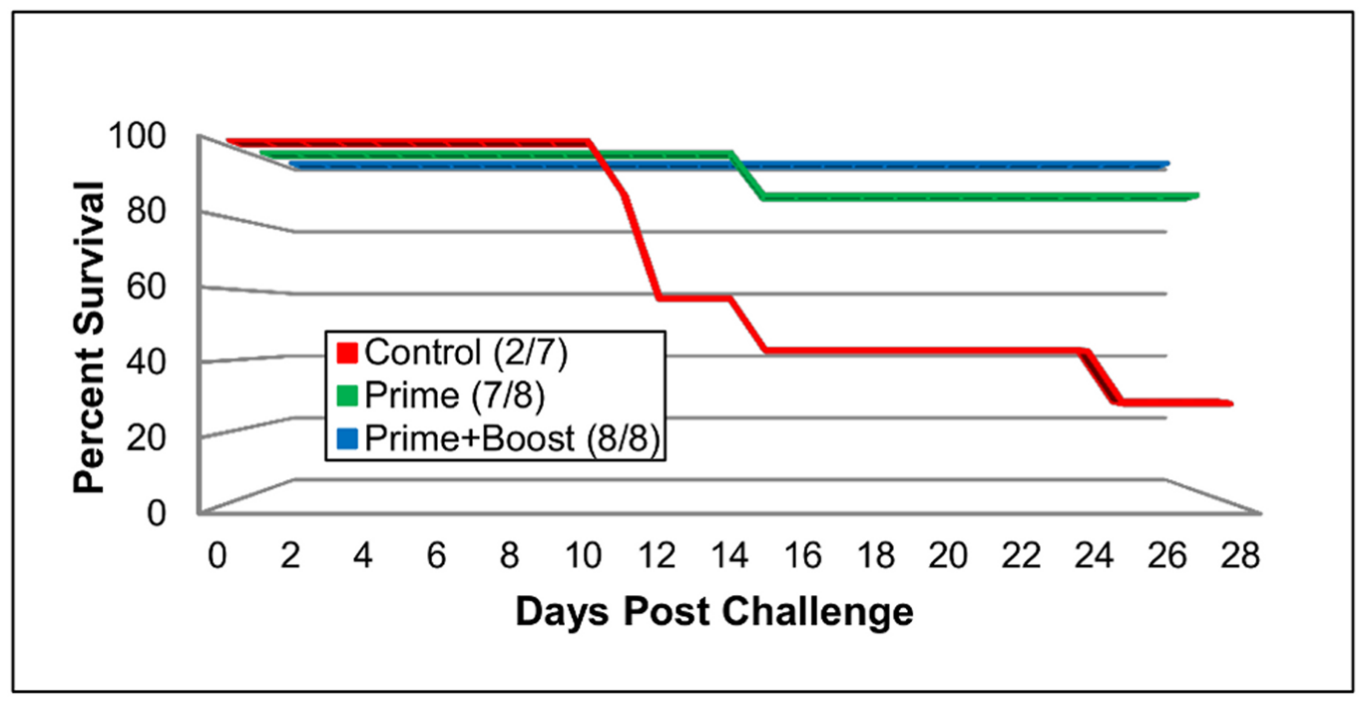
Percent survival of AG129 mice after vaccination with an adjuvanted inactivated EV71 vaccine. Five week old AG129 mice received i.m. injections of an adjuvanted inactivated EV71 vaccine. Control mice received PBS (same route and volume). Three weeks after prime, an optional boost of the vaccine was delivered (same route and volume). Two weeks after boost, all mice were challenged i.p. with 1.3×10^5^ TCID_50_/ml of the mouse adapted EV71. After the onset of clinical signs of disease, mice were euthanized.

**Figure 7:**
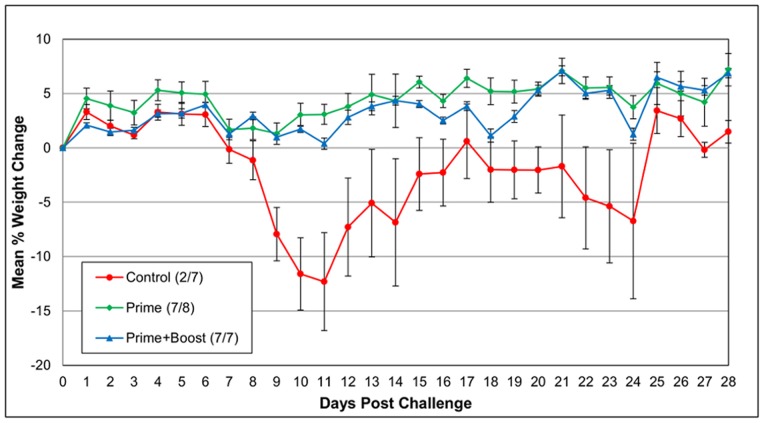
Mean percent weight change of mice after challenge with mouse adapted EV71 in active immunization. Five week old AG129 mice received i.m. injections of an adjuvanted inactivated vaccine of EV71 currently going through clinical trials. Control mice received PBS (same route and volume). Three weeks after prime, an optional boost of the vaccine was delivered (same route and dose). Two weeks after boost, all mice were challenged i.p. with 1.3×10^5^ TCID_50_/ml of the mouse adapted EV71. Mice were monitored for four weeks after challenge for clinical signs of disease including neurological complications and weight loss.

In a second study, the mouse adapted EV71 strain was used to evaluate the protection conferred by the EV71 inactivated vaccine. Three groups (n = 7–8) of 10 week-old AG129 mice were challenged i.p. with the mouse adapted strain on day 35 post prime. All animals that received a prime and booster injection of the vaccine survived, while one of the mice in the prime only group died on day 14 post challenge (Fig. 6). All control mice developed clinical signs of disease, lost weight, and five out of seven animals were euthanized between days 10 and 24 post challenge (Fig. 6). Statistically significant differences in weight loss from both the prime and prime-and-boost groups compared to the controls was detected between days 9–15 and 19–23 post challenge (P≤0.05). One mouse in the prime only group lost 20% of its starting weight, while the rest in this group and all mice in the prime-and-boost group either maintained or gained weight over the period of the study (Fig. 7).

### 3.4 Immunogenicity of EV71 Vaccine in AG129 Mice

**Figure 8:**
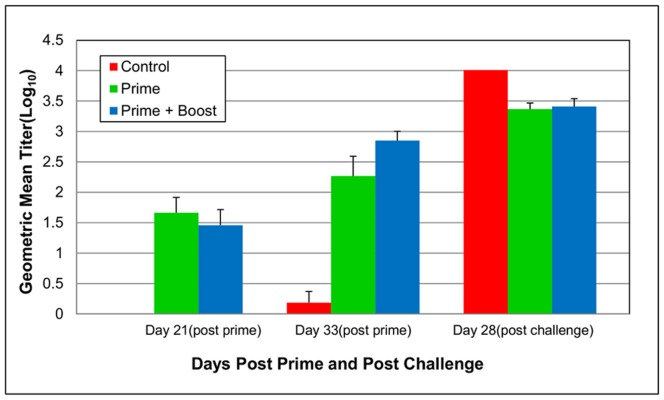
Neutralizing antibody responses against EV71 following immunization with an inactivated adjuvanted vaccine. Blood samples were collected at day 21 and 33 post prime and on day 28 post challenge during active immunization study. They were used to measure the neutralizing antibody responses to EV71 by TCID_50_. Bars represent geometric mean titer (GMT) ± standard error (SE). When comparing the GMT from days 21 to 33 post prime, the group of mice which received a booster vaccination had a 25 times greater increase in titer (P<0.01), whereas the prime only group had an increase of four times.

The geometric mean titers (GMT) of neutralizing antibodies elicited by the inactivated vaccine against the homologous strain of EV71 are shown in Fig. 8. On day 33 post prime, the GMT in adult AG129 mice which received a booster vaccination was four times greater than mice which received only a single dose of the vaccine. When comparing the GMT from days 21 to 33 post prime, the group of mice which received a booster vaccination had a 25 times greater increase in titer (P<0.01), whereas the prime only group had an increase of merely four times. All control mice before challenge had significantly lower GMT of equal or less than 20 (P≤0.05). Surprisingly, the two AG129 control mice which survived challenge had a significantly greater GMT four weeks after challenge than both groups of vaccinated mice (P≤0.05).

### 3.5 Histopathology

**Figure 9:**
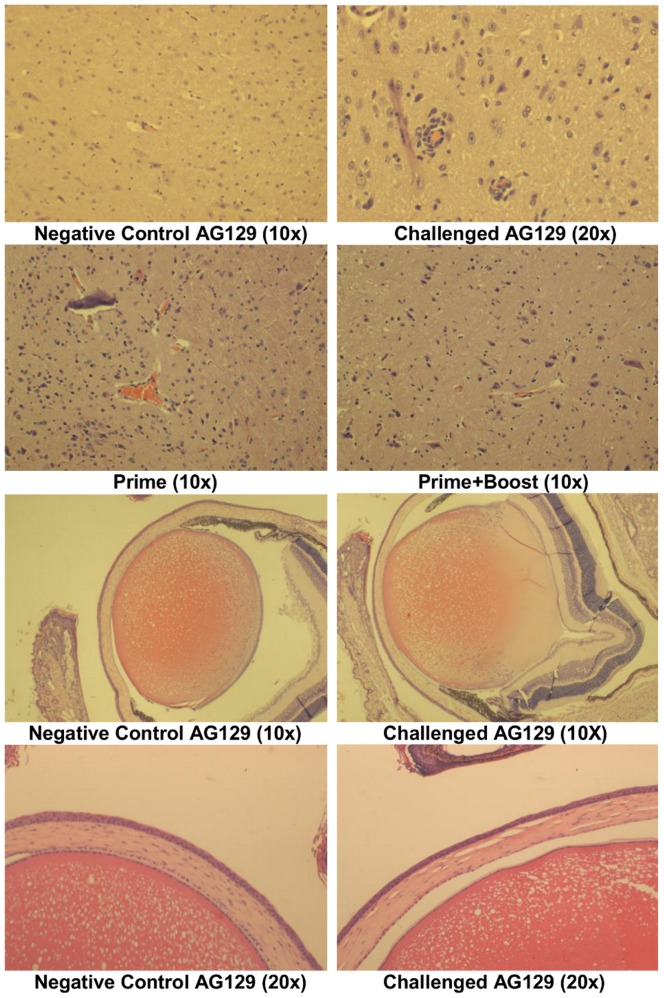
Histopathology of brain stem and eyes of AG129 mice in the active immunization study. Five week old AG129 mice received i.m. injections of an adjuvanted inactivated vaccine of EV71. Control mice received PBS (same route and volume). Three weeks after prime, an optional boost of the vaccine was delivered (same route and volume). Two weeks after boost, all mice were challenged i.p. with 1.3×10^5^ TCID_50_/ml of the mouse adapted EV71. Perivascular cuffing was observed in the brain stem of challenged animals along with gliosis. Prime group had gliosis while the prime-and-boost group was similar to negative control. Lens and corneal edema was observed in the eyes of challenged animals.

Clinical signs of mild encephalitis were observed in all of the adult AG129 mice challenged with the mouse adapted EV71 strain. Perivascular cuffing and gliosis were seen in the brain stem (Fig. 9). Mice that received a prime only of the inactivated vaccine showed signs of mild infection with gliosis in the brain stem. The mice which received prime-and-boost vaccinations, as well as the PBS control mice showed no clinical signs of disease in the brain stem. Histological studies done on brain stems from the comparison study showed signs of mild encephalitis in the AG129 mice and one of the A129 mice challenged with the mouse adapted strain of EV71. PBS and parental strain control groups had no lesions in the brain (pictures not shown). The eye globe of infected adult AG129 mice showed edema in the lens and cornea (Fig. 9). No lesions were observed in any of the vaccinated mice or adult A129 mice. The hearts, livers, intestines, lungs, spleens, kidneys, and hind leg muscles of all mice were screened for clinical signs of disease. No histological changes were observed in any of these organs from all groups of mice used throughout these studies.

### 3.6 Viremia

**Figure 10:**
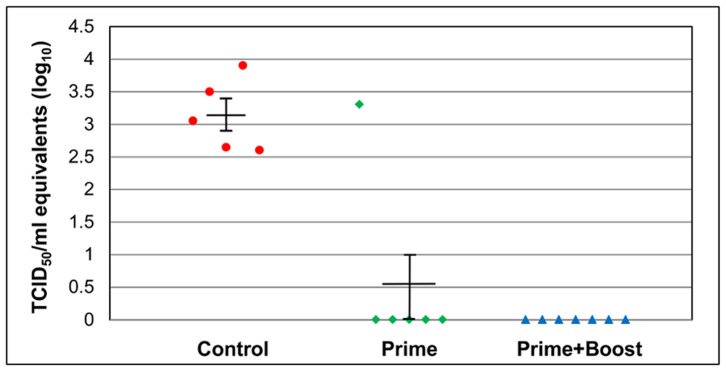
Viremia of AG129 mice on day three post challenge during active immunization study. Groups of AG129 mice were bled on days 3 and 7 post challenge in active immunization study to test for viremia by SYBR Green real time PCR. The mice were challenged i.p. with 1.3×10^5^ TCID_50_/ml of the mouse adapted EV71. Day 3 showed statistically significant viremia levels in the unvaccinated control mice when compared to the prime and prime-and-boost group (P≤0.05). Day 7 showed no signs of viremia in any of the groups.

Significant differences in the levels of viremia between control and vaccinated mice were detected in serum samples collected on day three post challenge (P≤0.05). None of the mice in the prime-and-boost group had detectable viremia on day 3 post challenge (Fig. 10). Interestingly, the mouse in the prime only group that became sick and was euthanized following challenge also showed positive viremia results. When tested at day seven post challenge, all serum samples were negative by PCR indicating that no viremia was present at that time point (data not shown).

## Discussion

This work describes the generation of a mouse adapted EV71 virus and the development of an animal model using adult 10 week-old IFN deficient AG129 mice. The lack of an adult model has hampered the efforts to evaluate the efficacy of candidate vaccines against EV71 as well as study its pathogenesis. Here, we demonstrate how we overcame these obstacles with the combined use of our EV71 mouse adapted strain and adult AG129 mice. A side by side comparison study using 10 week-old A129 and AG129 mice highlighted the importance of IFN-γ in controlling EV71 infections in the absence of IFN-α/β. It also demonstrated that adaptation of EV71 was necessary as mortality was not observed following the inoculation of the parental strain in age matched adult mice. Furthermore, active immunization and passive transfer studies in our IFN deficient mouse model demonstrated the protective capacity of a candidate inactivated EV71 vaccine and the importance of antibodies in controlling EV71 infections.

Previously, strains of outbred and IFN deficient mice have been tested as models for EV71 vaccine efficacy and pathogenicity studies [14], [15], [20], [21], [22], [23]. Susceptibility of AG129 mice to EV71 infection was age-dependent with resistance being observed after two weeks of age [12]. In other studies, mouse adapted strains of EV71 were developed through serial passages of parental strains in Institute of Cancer Research (ICR) or BALB/c newborn mice and cell culture [20], [22], [23], [24]. Serial passages included isolating EV71 from the brain tissue of newborn ICR mice and then passaging it through cell culture to increase the virulence of EV71 in mice [20], [23]. Similar methods have also been done using muscle tissue from ICR and BALB/c mice [22], [24]. Unfortunately, all these models did not increase the age of susceptible mice past two weeks. Susceptibility was also dose-dependent requiring a high viral titer. Need for such titers limits the use of these models and pose problems in testing the efficacy of potential vaccines.

Our model combined the use of IFN deficient mice as well as adaptation through serial brain passages, resulting in adult mice succumbing to disease. The mouse adapted strain developed using a B2 sub-genotype strain of EV71 shows central nervous system (CNS) clinical signs as seen in previous mouse studies [14], [23] and human cases [2]. They include limb paralysis, hunched back, loss of control and balance, eye irritation, and weight loss. Histological findings showed lens and corneal edema. Eye irritation has been a clear indication of systemic infections and has been seen in closely related virus, including Enterovirus 70 and Coxsackievirus A24 as acute hemorrhagic conjunctivitis [25]. In our adult AG129 model, clinical signs of mild encephalitis were also observed. Lesions of perivascular cuffing and gliosis were detected in the brain. Similar clinical signs and lesions have been reported in humans following EV71 infections [26].

The systemic spread of EV71 to the CNS has been reported in previous studies with both the bloodstream and neuronal pathways involved [20], [27]. High levels of viremia and viral tissue loads have been seen on day three post-infection in previous mouse studies [20], [27]. Here, we report similar findings with an increase in viremia on day 3 post challenge. Studies indicate that EV71 enters the CNS through retrograde axonal transport, although some work also indicate access through the blood brain barrier [27]. It has been shown with poliovirus that viremia results in viral spread and replication in many organs including limb muscle and provides a source of virus for the CNS [28]. Similar studies for EV71 in mice indicate viral replication in the limb muscles followed by spread to the brainstem via the anterior horn motor neurons of the spinal cord [14], [20], [27]. Based on the clinical signs, viremia, and histological findings, it is possible that a similar neurotropic spread route is occurring following i.p. infections of the adult AG129 mice. While i.p. infections were shown to allow the virus to enter the CNS in adult AG129 mice, it is important to determine the susceptibility of our mouse model following a more natural mode of infection such as the oral route, which is the natural route of infection in humans [2]. We recently observed 67% mortality in one week-old AG129 mice that were orally infected with our mouse adapted EV71 strain (data not shown).

Our comparative study showcased the importance of using AG129 mice (lacking IFN-α/β and γ receptors) in order to demonstrate susceptibility of EV71 disease in adult mice. It also suggests that IFN-γ can provide protection to EV71 and highlights the importance of both type I and IFN-γ in controlling EV71 infections. Adult A129 (lacking only α/β IFN receptors) mice displayed resistance to infection. A previous study reported the importance of type I IFN as a major innate defense mechanism and their role in controlling against EV71 infections [16]. Pre-treatment with a neutralizing antibody to IFN-α/β dramatically increased the susceptibility of ICR mice to EV71 [16]. Past results have also cited high levels of IFN-γ being associated with neurological complications in EV71 patients [29]. However, our findings have shown how the presence of IFN-γ in A129 mice can contribute to controlling disease. IFN-γ has been shown to induce type I nitric oxide synthase (NOS) in neurons and prevent the spread of vesicular stomatitis virus to the CNS *in vivo* and stop the replication of human poliovirus *in vitro* [30]. The lack of α/β and γ IFN in the AG129 mice made it possible to increase the age of susceptibility to adult mice.

The usefulness of IFN deficient mice in the development of an animal model has been demonstrated in past studies [11], [12], [14]. They have allowed for pathogenesis and host immune response studies. Despite the lack of a functional IFN response, these mice mount an immune response to viruses and various antigens [31]. Our mouse model was suitable for testing the efficacy of a purified inactivated EV71 vaccine candidate. Previous data revealed that the alum adjuvanted EV71 candidate vaccine can elicit high levels of neutralizing antibodies in mice, rats, and rabbits and sustain those levels over a long period of time [unpublished data]. Through both passive transfer and active immunization studies these high levels of neutralizing antibodies produced from the vaccine were shown to be protective in our model. Many studies have shown the importance of neutralizing antibodies in protecting against an EV71 infection [14], [15], [21]. Neutralizing antibodies prevent viremia and the spread of the virus into varying organs and the CNS with a single dose of an inactivated vaccine [21], [32]. The immune sera used in our studies follows these results, showing antibodies produced from the inactivated EV71 vaccine are enough to stop viremia and onset of neurological signs of infection. Neutralizing antibodies have also been shown to be cross reactive to other genotypes of EV71 [15], but further studies would have to be done to conclude this using the inactivated vaccine used in our studies.

Active immunization studies have been limited and performed in 1 day-old ICR mice. These mice have immature immune systems and full vaccine induced immune responses are not achieved prior to EV71 challenge [33]. Our active immunization study was performed in adult AG129 mice that received EV71 challenge five weeks after the initial prime injection. The vaccine was completely protective when both a prime and boost was administered to the mice. These data are consistent with findings from the recent phase I clinical trial in humans. One hundred percent of the humans which received both a prime and boost of the vaccine in humans had a significant increase in EV71 immune responses after immunization, which may be a sign of protection against infection [unpublished data].

We hypothesized that the adaptation of EV71 in adult IFN deficient mice resulted in mutations in viral genome. Based on previous adaptation experiments, it is likely that these changes occurred in the VP1–VP4 genes, involved in capsid formation, or 2C genes, involved in viral encapsulation [21], [22], [23], [24]. In many adaptation processes the most common amino acid changes were found in either the VP1, VP2, or both when compared to the parental strain [21], [22], [23], [24]. It is likely that these mutations lead to changes in the viral receptor binding region. Recent studies have shown the VP1 gene as the target for the human scavenger receptor class B2 (SCARB2) which is the main cellular receptor for EV71 in humans [34]. Other studies have also found amino acid changes occurring in the 2C region [22], [23]. We are currently making infectious cDNA clones of our parental and mouse adapted strain in order to map the genome changes that occurred during the mouse adaptation of the EV71 virus used in these studies.

In conclusion, we have successfully demonstrated the importance of an adult animal model that can significantly expand the knowledge and understanding of the pathogenesis of EV71 virus. By adapting a B2-subgenotype of EV71 to adult IFN deficient mice we were able to use this model in passive transfer and active immunization studies as well as to explore the immune response of mice to EV71 infection. A similar approach can be used to develop models for other important non-polio enteroviruses such as Coxsackie A6 and A16 viruses. Future studies can also be done to determine the role of cell mediated immunity in protection from disease and more strains can be adapted in order to test whether vaccines can protect from heterologous challenge with different strains of EV71. EV71 outbreaks are occurring at a rapid rate and there is an urgent need for a vaccine or antiviral. Our AG129 mouse model can be a new tool to expand our knowledge of EV71 and achieve this goal.

## References

[1] Huang CC, Liu CC, Chang YC, Chen CY, Wang ST, et al (1999) Neurologic complications in children with enterovirus 71 infection. N Engl J Med 341: 936–942. 10498488 10.1056/NEJM199909233411302

[2] Solomon T, Lewthwaite P, Perera D, Cardosa MJ, McMinn P, et al (2010) Virology, epidemiology, pathogenesis, and control of enterovirus 71. Lancet Infect Dis 10: 778–790. 20961813 10.1016/S1473-3099(10)70194-8

[3] McMinn PC (2002) An overview of the evolution of enterovirus 71 and its clinical and public health significance. FEMS Microbiol Rev 26: 91–107. 12007645 10.1111/j.1574-6976.2002.tb00601.x

[4] Zeng M, El Khatib NF, Tu S, Ren P, Xu S, et al (2012) Seroepidemiology of Enterovirus 71 infection prior to the 2011 season in children in Shanghai. J Clin Virol 53: 285–289. 22265829 10.1016/j.jcv.2011.12.025

[5] (2012) WPRO Hand, Foot and Mouth Disease Situation Update 2011–24 July 2012. Western Pacific Regional Office of the World Health Organizaiton.

[6] Huang WC, Huang LM, Kao CL, Lu CY, Shao PL, et al (2012) Seroprevalence of enterovirus 71 and no evidence of crossprotection of enterovirus 71 antibody against the other enteroviruses in kindergarten children in Taipei city. J Microbiol Immunol Infect 45: 96–101. 22154997 10.1016/j.jmii.2011.09.025

[7] Tran CB, Nguyen HT, Phan HT, Tran NV, Wills B, et al (2011) The seroprevalence and seroincidence of enterovirus71 infection in infants and children in Ho Chi Minh City, Viet Nam. PLoS One 6: e21116. 21765891 10.1371/journal.pone.0021116 PMC3134465

[8] (2012) WHO Global Alert and Response (GAR) Undiagnosed illness in Cambodia-update 9 July 2012. World Health Organization.

[9] Shimizu H, Utama A, Yoshii K, Yoshida H, Yoneyama T, et al (1999) Enterovirus 71 from fatal and nonfatal cases of hand, foot and mouth disease epidemics in Malaysia, Japan and Taiwan in 1997–1998. Jpn J Infect Dis 52: 12–15. 10808253

[10] Li L, He Y, Yang H, Zhu J, Xu X, et al (2005) Genetic characteristics of human enterovirus 71 and coxsackievirus A16 circulating from 1999 to 2004 in Shenzhen, People’s Republic of China. J Clin Microbiol 43: 3835–3839. 16081920 10.1128/JCM.43.8.3835-3839.2005 PMC1233905

[11] Johnson AJ, Roehrig JT (1999) New mouse model for dengue virus vaccine testing. J Virol 73: 783–786. 9847388 10.1128/jvi.73.1.783-786.1999 PMC103889

[12] Brewoo JN, Kinney RM, Powell TD, Arguello JJ, Silengo SJ, et al (2012) Immunogenicity and efficacy of chimeric dengue vaccine (DENVax) formulations in interferon-deficient AG129 mice. Vaccine 30: 1513–1520. 22178727 10.1016/j.vaccine.2011.11.072 PMC4592107

[13] Khong WX, Foo DG, Trasti SL, Tan EL, Alonso S (2011) Sustained high levels of interleukin-6 contribute to the pathogenesis of enterovirus 71 in a neonate mouse model. J Virol 85: 3067–3076. 21228224 10.1128/JVI.01779-10 PMC3067852

[14] Khong WX, Yan B, Yeo H, Tan EL, Lee JJ, et al (2012) A non-mouse-adapted enterovirus 71 (EV71) strain exhibits neurotropism, causing neurological manifestations in a novel mouse model of EV71 infection. J Virol 86: 2121–2131. 22130542 10.1128/JVI.06103-11 PMC3302383

[15] Bek EJ, Hussain KM, Phuektes P, Kok CC, Gao Q, et al (2011) Formalin-inactivated vaccine provokes cross-protective immunity in a mouse model of human enterovirus 71 infection. Vaccine 29: 4829–4838. 21550375 10.1016/j.vaccine.2011.04.070

[16] Liu ML, Lee YP, Wang YF, Lei HY, Liu CC, et al (2005) Type I interferons protect mice against enterovirus 71 infection. J Gen Virol 86: 3263–3269. 16298971 10.1099/vir.0.81195-0

[17] Fensterl V, Sen GC (2009) Interferons and viral infections. Biofactors 35: 14–20. 19319841 10.1002/biof.6

[18] Reed LJaMHA (1938) A simple method of estimating fifty percent endpoints.. American Journal of Hygiene 27: 493–497.

[19] Rosner B (1995) Fundamentals of Biostatistics. CA: Duxbury Press.

[20] Chen YC, Yu CK, Wang YF, Liu CC, Su IJ, et al (2004) A murine oral enterovirus 71 infection model with central nervous system involvement. J Gen Virol 85: 69–77. 14718621 10.1099/vir.0.19423-0

[21] Ong KC, Devi S, Cardosa MJ, Wong KT (2010) Formaldehyde-inactivated whole-virus vaccine protects a murine model of enterovirus 71 encephalomyelitis against disease. J Virol 84: 661–665. 19864378 10.1128/JVI.00999-09 PMC2798416

[22] Wang W, Duo J, Liu J, Ma C, Zhang L, et al (2011) A mouse muscle-adapted enterovirus 71 strain with increased virulence in mice. Microbes Infect 13: 862–870. 21612764 10.1016/j.micinf.2011.04.004

[23] Wang YF, Chou CT, Lei HY, Liu CC, Wang SM, et al (2004) A mouse-adapted enterovirus 71 strain causes neurological disease in mice after oral infection. J Virol 78: 7916–7924. 15254164 10.1128/JVI.78.15.7916-7924.2004 PMC446098

[24] Zaini Z, Phuektes P, McMinn P (2012) Mouse adaptation of a sub-genogroup B5 strain of human enterovirus 71 is associated with a novel lysine to glutamic acid substitution at position 244 in protein VP1. Virus Res. 10.1016/j.virusres.2012.04.009 22575826

[25] Palacios G, Oberste MS (2005) Enteroviruses as agents of emerging infectious diseases. J Neurovirol 11: 424–433. 16287683 10.1080/13550280591002531

[26] Hao B, Gao D, Tang DW, Wang XG, Liu SP, et al.. (2012) [Distribution of human enterovirus 71 in brainstem of infants with brain stem encephalitis and infection mechanism]. Fa Yi Xue Za Zhi 28: 85–88, 91. 22619799

[27] Chen CS, Yao YC, Lin SC, Lee YP, Wang YF, et al (2007) Retrograde axonal transport: a major transmission route of enterovirus 71 in mice. J Virol 81: 8996–9003. 17567704 10.1128/JVI.00236-07 PMC1951457

[28] Ren R, Racaniello VR (1992) Poliovirus spreads from muscle to the central nervous system by neural pathways. J Infect Dis 166: 747–752. 1326581 10.1093/infdis/166.4.747

[29] Lin TY, Hsia SH, Huang YC, Wu CT, Chang LY (2003) Proinflammatory cytokine reactions in enterovirus 71 infections of the central nervous system. Clin Infect Dis 36: 269–274. 12539066 10.1086/345905

[30] Komatsu T, Bi Z, Reiss CS (1996) Interferon-gamma induced type I nitric oxide synthase activity inhibits viral replication in neurons. J Neuroimmunol 68: 101–108. 8784266 10.1016/0165-5728(96)00083-5

[31] van den Broek MF, Muller U, Huang S, Aguet M, Zinkernagel RM (1995) Antiviral defense in mice lacking both alpha/beta and gamma interferon receptors. J Virol 69: 4792–4796. 7609046 10.1128/jvi.69.8.4792-4796.1995 PMC189290

[32] Sutter RW, Pallansch MA, Sawyer LA, Cochi SL, Hadler SC (1995) Defining surrogate serologic tests with respect to predicting protective vaccine efficacy: poliovirus vaccination. Ann N Y Acad Sci 754: 289–299. 7625665 10.1111/j.1749-6632.1995.tb44462.x

[33] Wu TC, Wang YF, Lee YP, Wang JR, Liu CC, et al (2007) Immunity to avirulent enterovirus 71 and coxsackie A16 virus protects against enterovirus 71 infection in mice. J Virol 81: 10310–10315. 17626076 10.1128/JVI.00372-07 PMC2045469

[34] Chen P, Song Z, Qi Y, Feng X, Xu N, et al (2012) Molecular determinants of enterovirus 71 viral entry: cleft around GLN-172 on VP1 protein interacts with variable region on scavenge receptor B 2. J Biol Chem 287: 6406–6420. 22219187 10.1074/jbc.M111.301622 PMC3307280

